# Unconscious Malingering in Childhood

**Published:** 1921-08-20

**Authors:** 


### THE PUBLIC WELL-BEING.

### Unconscious Malingering in Childhood.

While the health of the nation continues to improve, while the birth-rate rises and the death-rate falls, while the infant-mortality rate is the lowest recorded, the annual report for 1920 of Sir George Newman, Chief Medical Officer of the Ministry of Health, exposes a very serious average sickness-rate among the children of the nation. A great mass of relatively trivial symptoms come under the notice of the doctors through whose hands the children of school age periodically pass. If neglected, these symptoms sometimes persist and lead to more serious conditions. They are often extremely baffling, for they occur in children who have to all appearance been well-born, and with whose environment there is little fault to be found.

We have before us the report of the School Medical Officer of East Sussex County Council for 1920, and we note that the number of individual children inspected by the medical officers totalled 11,141. Out of this number (and excluding those who were referred on account of uncleanliness or defective clothing or foot-gear) 1,760 were referred for treatment and 1,329 required to be kept under observation, making a total of 3,089 ailing children. Thus rather more than a quarter—to be precise, 27 per cent.—of the whole population of East Sussex under instruction in elementary schools presented symptoms of deterioration in health. As regards secondary schools, the conditions are distinctly worse, for out of 517 children inspected 147 were referred for treatment and fifty-seven required to be kept under observation.

It would be difficult to find healthier conditions in the United Kingdom than those which surround the children of East Sussex. The problem for preventive medicine, working hand-in-hand with the educational authorities, is to discover the causes which operate to break down resistance to disease in so large a proportion of the young.

There is a danger, which we should hesitate to describe as negligible, that the present system of hunting down early symptoms which may reveal a lack of resistance to disease confers too much prominence on the ailing child, and acts as an incentive to the morbid or lazy to play into the hands of the anxious mother. There is a tendency, even among healthy children, to enjoy the kudos conferred by having "something the matter" with them. Kipling, with a shrewd memory or power of observation, presents the Brushwood boy to us at the age of three gloating over his cut finger and the risk that he may bleed to death. To be conspicuous is the first instinct, and the ailing child not only secures this prize but many others, constant escape from school, delicacies and toys to make up, holidays for change of air, the best that life can afford in his little environment.

It is strange that no counter-attraction in favour of health has yet been set stirring among the children. The prizes rarely offered for a year's unbroken attendance at school come nearest to such an incentive; but the condition is too unyielding to affect a large number. A regular festivity or school-treat at the end of each term for those only who had not missed more than one attendance, and prizes of a similar nature at the close of the year, might effect much. Really well-organised games would do even more. But while it is certain that the pleasures of health are their own reward, it will not do to leave the distinction of ill-health without a rival. The athlete will always be comparatively rare. We plead for some genial appreciation of the healthy, hardy stock, indifferent to weather, inured to fatigue—in a word, resistant to disease. It is just possible that preventive medicine may be overlooking the need to lay more stress on health than on the onset of disease.

